# Dentists and new digital appliances ‐ to buy or delay until the next model?

**DOI:** 10.1002/cre2.56

**Published:** 2016-12-28

**Authors:** Asbjorn Jokstad

**Affiliations:** ^1^ Institute of Clinical Dentistry, Faculty of Health of Health Sciences UiT, The Arctic University of Norway Norway

Three years after dental school graduation in 1979, I signed up for graduate studies in information technology at the University of Oslo. The impetus was the Norwegian success story variant of the Finnish “Nokia” cell phone chronicle; a company named “Norsk Data” who managed for a short while in the 80ies to dominate the global market for what was then termed “mini‐computers”. The studies didn't secure me a job in Norsk Data, but landed me instead at the Department of Oral Anatomy to work with their newly acquired digital electron microscopes. Serendipity brought me one year later to the Nordic Institute for Dental Materials (NIOM), when their central computer crashed and they desperately needed a computer geek to do the tedious reconstruction of corrupted data files.

I have maintained an interest for applications of computer hardware and software applications in dental research and clinical practice. It may perhaps have been to the chagrin of my students, whom I consistently implored to comprehend instead of only making use of computer‐assisted tools and digital appliances for particular purposes. We have probably all experienced repeatedly that the latest generations of digital products on the market are not guaranteed improvements of previous products. The matter may not be so important if it is a consumer product for personal use, because you will only have wasted your money if you end up buying a lemon. But what if you plan to purchase a new computer‐assisted technology for use in a patient clinic with the aspiration to provide safer and speedier care with less inconvenience and more predictable diagnoses and treatment outcomes? Try to recollect when during your DDS training you learned how to critically appraise the potentials and the flaws of computer technologies that are being promoted as a solution for you and your patients' needs? Do you know which relevant questions to ask about e.g., bug fixes since the product release, and practical limitations of use or type of support etc. or is selling digital appliances to dentists that display a positive interest like selling candy to kids?

The editor of Journal of Oral Rehabilitation recently commissioned a small group of colleagues to review various aspects of the use of digital technologies in oral rehabilitation. Undersigned concludes that two conditions apply in the computing industry, which pertains also to digital appliances for use in dentistry (1). The first is that “Moore's law or prediction” made in 1965 still rules. In short, twice as many semiconductors can be placed on an integrated electronic circuit every second year. The effect is a continuous increase of the capacity of microprocessors in terms of speed and memory and invariably also in better stability and lower price versus performance. The second is that innovative software programs will harness these improvements in performance. The net effect is that the product life cycle of a new digital product is worryingly unknown, but is in general short‐lived. As a consequence, manufacturers choose different strategies to attain the return on their R&D investments as fast as possible. Still, it is important that clinicians can judge the merits of a new digital product based on clinically meaningful information, and not only on technological verbiage. Unfortunately, of the approximately 225 digital products that were available on the market per November 2016, only about one third has been described in a clinical human study of n+1 (1). Moreover, while the great majority of digital products have minimal or no clinical documentation at all, the products from a distinct minority of manufacturers frequently appear in more or less scientific reports. One inference is that the clinician should stick to the clinically validated products, alternatively seek to improve their knowledge and understanding of benefits and drawbacks of current computer‐assisted tools and digital appliances. Unfortunately, it is difficult to find independent and objective information on the internet. Be aware that searching the internet by use of a search engine profiles you, (or rather the IP address of your computer), causing a stream of advertisements over the following weeks and months from the companies that have purchased such services from the firm that owns the search engine. To stop building the capital base of the search engine owners, the alternative is to use the duckduckgo.com search engine or the Tor browser.

During the preparation of my review of digital appliances in oral rehabilitation I often reflected on how the careers of peers in my generation have been influenced by what the computing industry has brought to the market. These are not original thoughts and have reverberated ever since the luddites went rampaged two centuries ago after being rendered jobless by the invention of cotton milling machines. An economist today will likely be able to write a persuasive and optimistic essay on why the computer technology progress is not only inevitable, but also good for society. However, the dark side will still remain, which is that new technologies will often also signify job layoffs. If this line of thought appears too negative, the reader is advised to examine the job lookout guidance over the last ten years for the future needs of dental laboratory technicians. Once again in history, highly skilled workers are being replaced by milling machines requiring fewer working hands, although this time not for spinning cotton but for manufacturing dental devices. Time will show whether independent dental technician laboratories can remain as a sustainable business model, or whether the continuous developments will force the dental technicians to return to work in individual clinics where their daily work tasks will be the designing and manufacturing of some dental devices locally in combination with finalizing devices manufactured by use of heavy machinery at large production centres.

1. Jokstad A. Computer‐assisted technologies used in oral rehabilitation and the clinical documentation of alleged advantages ‐ A systematic review. J Oral Rehabil 2017; in press.10.1111/joor.1248328109024

